# The Influence of Leather Type on Thermal and Smoke-Generating Properties

**DOI:** 10.3390/ma18020304

**Published:** 2025-01-10

**Authors:** Sebastian Staszko, Marzena Półka, Rafał Matuszkiewicz

**Affiliations:** Faculty of Safety Engineering and Civil Protection, Fire University, 52/54 Slowackiego Street, 01-629 Warsaw, Poland; rmatuszkiewicz@apoz.edu.pl

**Keywords:** fire safety engineering, natural leather, smoke generation

## Abstract

The main purpose of this article was to determine the smoke-generating and thermal properties of selected types of natural leather. Differences in the amount of smoke generated from the type of finish used in the technological processing of leather were observed. Research has shown that the burnt nubuck (367) sample with exposure at the heat flux intensity of 25 kW/m^2^ without the presence of a pilot burner flame achieved the highest value of the specific optical density D_s,max_. Comparable values, 312 and 297, were recorded for grain bovine leather and velour bovine leather, respectively; on the other hand, the lowest value of the tested parameter amounting to 220 was recorded for lacquered bovine leather. Tests executed with the use of thermogravimetric analysis show that except for nubuck leather, the start of thermal decomposition for all types of samples appears to be fairly similar and was found to be within the range of 275–282 °C. The highest value of thermal decomposition onset, i.e., 302 °C, was recorded for nubuck leather. The highest percentile of residues from thermal decomposition, i.e., 9%, was obtained for grain bovine leather. This implies that the least gaseous phase during its thermal decomposition in test conditions was generated by this type of leather. The highest average D_s,max_ value was obtained for nubuck (367), and decreased as follows: grain bovine leather (312) > velour bovine leather (297) > lacquered bovine leather (220).

## 1. Introduction

Leather is a very popular choice because of its unique qualities [1]. In prehistoric times, ancient tribes used to use this raw material to make outer clothing for their survival. Today, leather is not only used for clothing and accessories (clothing and haberdashery industry), but it is also widely used for furniture upholstery (furniture industry), car upholstery (automotive industry), and also for interior finishing elements, such as walls in diverse facilities, e.g., hotels (construction industry). The increased production of leather goods and the wide range of applications for leather demands experimental research and the application of new technologies, not only to improve performance but also to enhance fire safety [2,3]. Moreover, there are many factors that shape leather and also influence its technological purpose. These factors include the animal’s breed, species, sex, age, diet, climatic conditions, living conditions, and the animal’s individual characteristics [4,5,6].

Genuine leather is a natural product that comes from the skin of animals. It is produced by removing the hair from the animal’s skin and then treating it with chemicals and oils. This consists of several chemical and mechanical procedures, which as a rule comprise pre-tanning, tanning, and post-tanning [7,8]. These processes make the leather more elastic and less prone to decay. Unlike synthetics, natural leathers contain collagen fibres in their structure, which means that products made from them have the ability to wick away moisture and, to a limited extent, allow airflow [9].

However, studies have shown that the leather industry appears to be one of the most polluting industries because of its production of massive amounts of waste. According to estimations, the processing of leather produces 200 times more waste volume than the overall production of the product [10].

Progress in sustainable technologies for leather waste valorisation is a solution for the circular economy [10]. The literature presents a number of methods for managing waste from leather production, which include, among others, tanning waste materials or energy, and also chemical, thermal, and biological techniques. Several secondary feedstocks may be recovered with the use of those processes, including chromium, nutrients, collagen hydrolysate, fat, biogas, and anaerobic digestate, which can then be reused in other industrial processes. Processes and operations required for processing hides and skins are commonly notorious for generating unpleasant odour [11,12,13,14].

Thermal processes, such as incineration, are increasingly used to dispose of municipal, industrial, or hazardous waste, including leather. Controlled incineration of leather production waste enables effective reduction in the volume of waste, leaving only ashes. The simultaneous recovery of energy contained in the waste mass is also feasible, which, given the rising prices of conventional energy sources (oil, gas), is not insignificant. In accordance with [15], the ash that remained in the furnace contained 36% Cr_2_O_3_ contaminated with NaCl. The analysis also revealed that the chromium(VI) content of the ash was only 260 ppm, indicating that only a small amount of the chromium present in the waste is oxidised during the process.

TG-FTIR was used in this research to investigate the thermal behaviour of pyrolysis and combustion of leather waste. Once the temperature of 330 °C was reached, differences were observed in the TG/DTG profiles between pyrolysis and combustion. According to the kinetic parameters of pyrolysis and combustion, the thermal decomposition of leather waste may become catalysed by oxygen under high temperatures. During pyrolysis, CO_2_, NH_3_, HCN, HNCO, CO, acids, and hydrocarbons were detected, with NH_3_ being the most important nitrogen-containing product. It was ascertained that NH_3_ and HNCO were the principal products that contain nitrogen during low-temperature combustion, while CO_2_ was found to be the dominant product above 400 °C. The major part of the thermal removal of nitrogen compounds from leather waste has taken place at lower temperatures, with nitrogen oxides not being identified because of their brief residence time in the atmosphere. [16]. A thermogravimetric analysis was carried out on the incineration of plastic (PVC), rubber (TR), and leather (L) in different atmospheres (80% N_2_/20%O_2_, 80% CO_2_/20%O_2_, 70% CO_2_/30%O_2_, 60% CO_2_/40%O_2_, 50%CO_2_/50%O_2_). Irrespective of the specific material or mixture used, the substitution of N_2_ for CO_2_ alone yielded lower mass loss rates, altered reactions taking place above 600 °C, and inferior combustion. The oxygen-enriched combustion technology may have mitigated the inhibitory effects to a certain extent. The impact of the mixing ratio on the rate of the fourth peak weight loss and burnout was dependent on the concrete atmosphere [17]. The burnout of Leather-PVC (L-PVC) blends in an atmosphere of 80% N_2_/20%O_2_ was inconsistent with the weighted average (the residual weight of 30% PVC/70%L was found to be 26.76% higher than that of pure PVC or L), which indicate that the interaction of PVC and L impeded the burnout [17].

Thermal decomposition and combustion products released from leather may contain readily flammable hazardous organic substances, including, for example, compounds left from processes of fat liquefaction, dyeing, and finishing processes of those materials [18]. The impact of various operations involving leather processing (such as tanning, oiling, etc.) and also of additives used, such as tannins and oiling, retanning agents, and those that reduce flammability, on the quality of materials produced, are presented in this study [19]. Further research [20,21] involves studies on the effect of tanning, which includes the use of a variety of tannins (chromic tannin, i.e., a tannin based on glutaric aldehyde and vegetal tanning agents) on the flammability of leather.

Given its structure and composition, leather offers an inherent and comparatively high resistance to fire, yet is, in general, a flammable material that can ignite both without and with a flame [18,19]. A fire can be started if the leather is not appropriately stored, in case of the lack of due care for fire protection or simply due to plain recklessness, and the heat and smoke generated released from the leather clearly impact the fire dynamics prevailing within building structures. It is therefore important to be familiar with the thermal and smoke-generated properties of leather in order to be able to ensure enhanced fire safety [22].

Smoke is generated in almost all fires and is a major source of hazard to human health and life. Reduced visibility caused by the absorption and scattering of light by the smoke generated, as well as the visual and respiratory effects of the toxic gases contained in the products of combustion, can cause disorientation in people fleeing from the objects involved in a fire. Smoke generated during the combustion of leather is characterised by varying intensity and composition. The smoke-generated content depends primarily on the material of origin and the conditions of thermal decomposition. Smoke generated is produced as a result of incomplete flame combustion and during flameless combustion. The smoke generated during this reaction is similar to that produced during the combustion of hydrocarbons. During combustion, the compounds are heated to temperatures at which thermal decomposition takes place and volatile components are released [23]. The heated high-molecular-weight volatile fractions become mixed with cold air, which causes them to precipitate as liquid droplets and tars with high boiling points. Dispersed in space, the particles with a diameter of about 1 micron, due to the lack of airflow, form a cloud-like suspension. As they settle, an oil residue is formed. During flame combustion, the smoke generated is primarily composed of solid particles, i.e., soot. The volatile components in the flame can be involved in a number of decomposition reactions that lead to the formation of new molecular structures. These include polycyclic hydrocarbons and polyacetylenes, which are usually the starting point for the formation of soot. Soot particles with a diameter of between 10 and 100 nm can be oxidised inside the flame, while if the temperature and oxygen concentration are not high enough, the soot particles tend to increase their agglomeration size. This produces larger soot particles that fall due to gravitational forces. There are various parameters that describe the properties of smoke generated, these include: type of medium, particle size, shape of aggregates, number of components and mass concentration, electrical charge value, and degree of aggregation [24,25,26].

The aim of this study was to analyse the thermal and smoke parameters of typical leathers that are part of interior finishes and furnishings in order to determine the fire risk. The selected thermal exposures in this study simulate the first phase of fire development (onset)—an external heat flux incident on the material of 25 kW/m^2^ with and without flame.

## 2. Materials and Methods

### 2.1. Materials

The leathers tested were commercially available finished products and were produced in the Production and Trading Company in Poland and they were made conventionally. All the materials studied were tanned from bovine leathers (mature cows) and tanned using different technologies. The leathers tested are used for footwear, upholstery, and accessories. Based on a market analysis, they are frequently purchased and these were deliberately chosen because they are part of the furnishings and interior finishes in residential buildings.

Four types of leather of animal origin were used in the experimental tests, namely:

- grain bovine leather;
- lacquered bovine leather;
- nubuck leather;
- velour bovine leather.

All the manufacturing reactions of the tested leathers were carried out in an aqueous environment. Hence, at the end of the leather processing, the contained water had to be removed. The drying technique (vacuum drying, dragging, etc.) has a significant impact on the leather properties and surface performance. Leather finishing is a group of surface operations aimed at improving the natural properties of leather. In the case of nubuck and velour, a solvent-based finish was used (colloid top, interlayer PU top, anionic solvent dye, synthetic wax, PU solvent, ethyl acetate, diacetone alcohol, hydrocarbon solvent), and for the finishing of the tested grain leather, polyurethane polymers were also used but mixed with natural oils. The binding agents (polymers) had a favorable effect on the opaque coating properties.

Data from the manufacturer indicate that the leathers were tanned with Cr(III) mainly in the form of chromium sulphate.

To finish the tested grain leather, mixtures of polyurethane polymers with natural oils were used.

The finishing process for velour and nubuck was complex. The first part of the process was the softening of the leather. It was carried out in rotating drums that were filled with fat emulsions with hygroscopic agents or aqueous silicone oil emulsions. In the second phase (dry phase), the fat was bound to the thin leather fibres. Then, using water and sodium bicarbonate, any fat that was no longer needed was rinsed out.

Lacquered leather was made from qualitatively inferior raw materials, as the applied layer of varnish covers most of the material’s imperfections. In the varnished leather in question, reactive polyurethane systems were used.

The elementary composition of tested samples was determined at the Institute of Organic Chemistry of the Polish Academy of Sciences in the Laboratory of Elemental Analysis and presented in Table 1. The analysis of the percentage of C, H, N, and S was performed in an automatic UNIcube analyser from the Elementary company in the Laboratory of Elementary Analysis. Moreover, the analysis of the percentage of sulphur content was mainly carried out using the Schőniger method. It consists of mineralising the substance in a flask with oxygen on a platinum catalyst and titrating with a standard solution against an indicator: the S—Schöniger method, which includes titration with a standard solution of Ba(ClO_4_)_2_.

The chrome-tanned leather in consideration was provided by a shoe factory in Poland. Nubuck has a layer of fine velvety fleece with short bristles in which individual fibres are visible. When running a finger over rough leather, a streaky effect is left on the leather, which is due to the change in direction of the bristles. Leather fleece is obtained by sanding the top of the leather (face) with increasingly fine sandpaper (of increasingly higher numbering). Lacquered bovine leather is the product of varnishing the top layer of leather several times using reactive polyurethane systems. They produce cross-linked lacquer coatings which make the final product high quality, durable in use, and with a beautiful, mirror-like surface.

### 2.2. Methods

#### 2.2.1. Thermogravimetric Method

Thermal analysis of the analysed leathers was performed using the dynamic technique according to PN-EN ISO 11358:2014 (Plastics. Thermogravimetry (TG) of polymers. General rules) [27], using a TA Instruments Q500 thermogravimetric analyser (New Castle, DE, USA). The method involved recording the change in mass of the test sample undergoing decomposition as a function of temperature at a programmed heating rate of 10 °C/min. The temperatures of the analysis ranged from 20 to 800/900 °C. The oxygen flow rate was 10 mL/min and the nitrogen flow rate was 90 mL/min. On the basis of the thermogravimetric curves obtained with the use of this method, the following parameters were established to compare the thermal stability of the materials tested:

- temperature of onset of thermal decomposition T0% [°C],
- temperature of the maximum rate of loss of specimen mass in the first phase of transformation [°C],
- temperature of the maximum rate of sample mass loss in transformation phase II [°C],
- temperature of 50% weight loss T50% [°C],
- maximum rate of weight loss in transformation phase I [%/min],
- maximum rate of weight loss in phase II of transformation [%/min],
- mass of the sample after thermal decomposition [mg]; [%].

Data obtained from the tests were recorded and presented graphically in the form of thermogravimetric curves using the TA Universal Analysis programme. On the basis of the authors’ previous experience and the device manufacturer’s documentation, the measurement accuracy was estimated at the following:

1 °C—for temperature measurement,

0.01 mg—for measuring the mass of the sample,

1 °C/min—for measuring the rate of sample mass loss.

#### 2.2.2. Single Smoke-Chamber Test

Using the single-chamber test method in accordance with EN ISO 5659-2:2017 [28], the smoke-generated production of the tested bovine leather samples was analysed at heat exposures of 25 and 50 kW/m^2^. A sample of the test material was placed in a special sample mounting pedestal located inside the chamber and subjected to a selected heat flux emitted by a conical infrared electric radiator. The alignment of the surface of the samples with respect to the surface of the radiator was parallel. Those conditions reproduced the flame combustion of the specimens in the first phase of fire development just prior to ignition. In this study, the optical properties of the smoke generated accumulated in the closed chamber, generated during the thermal decomposition and combustion of the material, and the qualitative components of the smoke generated were determined. Pursuant to the procedure presented in the standard [28] all smoke-generated measurements at the heat exposure analysed lasted 20 min and, once completed, the final results were recorded by a computer using software (Sychta Laboratorium Sp. J ISO5659, v.1.0).

The test chamber shall be constructed of laminated panels, the inner surfaces of which shall consist of porcelain enamelled metal of a thickness of not more than 1 mm or an equivalent coated metal that is resistant to chemicals and corrosion and allows easy cleaning. The internal dimensions of the chamber shall be 914 mm ± 3 mm in length, 914 mm ± 3 mm in height, and 610 mm ± 3 mm in depth. The chamber should have a front-mounted hinged door with an observation window and a removable opaque door cover for the window to prevent light from entering the chamber. The chamber door should occupy the entire side of the smoke chamber. There should be a safety panel in the chamber consisting of a sheet of aluminium foil not more than 0.04 mm thick with a minimum area of 80,600 mm^2^, fixed in such a way as to provide an airtight seal.

All specimens should be covered on the back, along the edges, and on the periphery of the front surface, leaving a central exposed area of 65 mm × 65 mm of the specimen, using a single sheet of aluminium foil (approximately 0.04 mm thick) with the matt side in contact with the specimen in standard [28].

**Figure 1:**
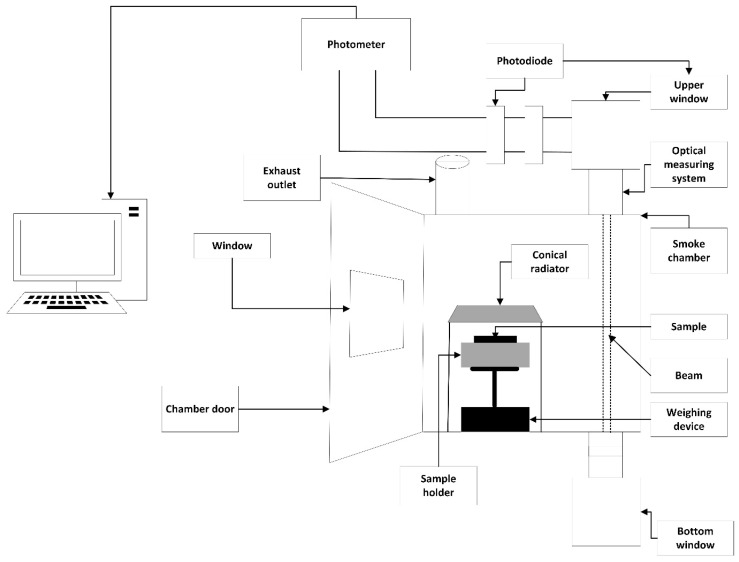
Single-compartment test bench diagram.

A schematic representation of the single-chamber test stand is shown in Figure 1.

In the single-chamber test, the specified optical density of the smoke is determined in accordance with Equation (1) [28], and the optical density of smoke in accordance with Equation (2)

(1) $$Ds=\frac{V_{k}}{A\cdot L}log\frac{T_{ro}}{T_{r}}=\frac{V_{k}\cdot D}{A\cdot L}$$

where:

D_s_—specified optical smoke density [-],
V_k_—volume of measurement smoke chamber [m^3^],
L—thickness of smoke layer [m],
A—active surface of sample [m^2^],
T_ro_—initial transmittance of light [%],
T_r_—light transmittance [%],
D—optical density of smoke [-].

(2) $$D=log\frac{I_{o}}{I}$$

where:

I—intensity of light that has passed through the smoke layer [-],
I_0_—light intensity that falls on the aerosol layer [-].

In accordance with the standard, other parameters were also recorded, such as the time until achievement of the maximum value of the specified optical smoke density (D_s,max_), the value of specific optical density after 4 min (D_s,4_), and the area under the curve of specific optical density during the first 4 min (V_OF4_).

V_OF4_ is a parameter indicating the amount of smoke released during the first 4 minutes of combustion of the sample. It is important in terms of the amount of smoke released. V_OF4_ is the area under the curve D_s_(t) as a function of time during the test period from t = 0 to t = 4, assuming a trapezoidal area and a finite time (t) of 1 min described by Equation (3), where D_s_(0) = 0, for t = 1 s:

(3) $$V_{OF4}=\int_{0}^{4}D_{s}\cdot dt$$

After reaching the D_s,max_, the values of specified optical smoke density decreased. This took place as a result of the vanishing of smoke due to coagulation, sedimentation, evaporation, and condensation on chamber walls.

## 3. Results

### 3.1. TG Analysis

**Figure 2:**
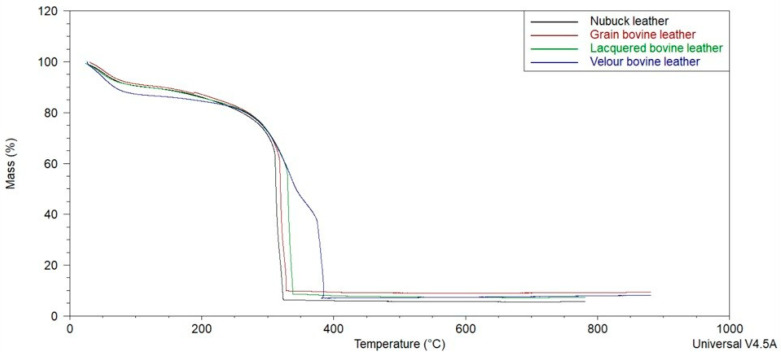
Obtained TG curves.

**Figure 3:**
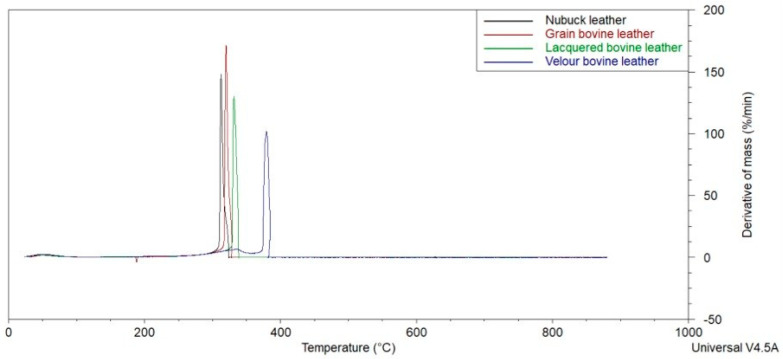
Obtained DTG curves.

Figure 2 and Figure 3 present the cumulative results of the obtained TG curves for the tested samples, as well as the curves of the sample mass loss rate over time.

Table 2 demonstrates the results of the TG analysis. The temperature of the onset of thermal decomposition of the sample is considered to be the point of intersection of the tangents to (a) the TG curve at the point where the derivative of the sample mass loss over time reaches its maximum and (b) the plateau after the evaporation of transient moisture.

Significant differences were observed in the course of thermal decomposition of the tested samples. Lacquered leather undergoes thermal decomposition at the lowest temperature of the tested samples, i.e., 275 °C, while nubuck requires temperatures above 300 °C. In an interval of about 20 °C, i.e., between 313 and 332 °C, the three samples (bovine, varnished, and nubuck) reach a maximum rate of sample weight loss. A significant difference was registered for the velour leather sample, for which this value was recorded at 382 °C. Meanwhile, for this velour leather sample, the residual value was found to be one of the lowest.

### 3.2. Single-Smoke Chamber Test Analysis

The average values of the smoke generated determined in the single-chamber test [28] are presented in Table 3.

The highest value of specific optical density was achieved by the burnt nubuck sample in all heat flux exposures analysed. These values were 367 (25 kW/m^2^ without the presence of a pilot burner flame), 146 (25 kW/m^2^ with the presence of a pilot burner flame), and 256 (50 kW/m^2^ without the presence of a pilot burner flame), respectively. Analysing the obtained values of D_s,4_ in the case of heat flux exposure of 25 kW/m^2^ with the presence of the pilot burner flame and 50 kW/m^2^ without the presence of the pilot burner flame, it was ascertained that the highest values were also obtained by the nubuck sample. The cause of this highest amount of smoke generated is the way in which this leather has been dressed and the highest carbon content of the leathers tested. This study shows that the introduction of a flame over the sample during testing to ignite the flammable products of thermal decomposition decreases the D_s,max_ value for all tested materials. The lowest D_s,max_ value was obtained for grain bovine leather at combustion with pilot flame (25 kW/m^2^).

## 4. Conclusions

In the scientific literature, no studies were found on the determination of the specific optical density of smoke in the single-chamber test in the thermal escapements analysed. The thermogravimetric analysis has shown that the beginning (onset) of thermal decomposition for all types of samples was within the range of 275–302 °C. Lacquered bovine leather exhibited the lowest value of the onset of thermal decomposition, i.e., 275 °C. Nubuck leather was found to be 27 °C higher than lacquered bovine leather, which additionally lost 50% of its weight at the lowest temperature among the tested materials (313 °C). The highest percentage value of the residue after thermal decomposition was obtained for bovine leather amounting to 9.1%. This indicates that this leather produces the least gas phase during its thermal decomposition under the test conditions. The lowest percentage of residues was recorded for nubuck leather, namely 5.5%. The difference between the remaining samples was small and ranged from 6.5 to 7.3%. The fastest weight loss was recorded for bovine leather, which amounted to 174%/min. The lowest value was recorded for velour leather, equalling 93.1%/min. In fact, it was this leather that underwent thermal decomposition within the largest temperature range (i.e., the longest one); the temperature of the end of thermal decomposition was 405 °C, and at the highest temperature, it had a maximum weight loss of 382 °C. Hence, this leather showed the highest smoke generated in the smoke test (D_s,max_ 367) at a heat exposure of 25 kW/m^2^ without a small external flame. A lower value of D_s,max_ than that for nubuck leather was recorded for grain bovine leather (312), velour bovine leather (297), and lacquered bovine leather (220). Lacquered bovine leather is characterized by the greatest dynamics of flame combustion. This type of leather ignited the fastest, because in the 4th second of the test when exposed to a heat flux of 25 kW/m^2^ with a pilot burner flame, while at a heat flux of 50 kW/m^2^ without the presence of a flame, ignition took place after 10 s. A similar ignition time was obtained for grain leather—9 s. The short time to ignition of the flammable gas phase of patent leather and grain leather reduced the D_s,max_ value; however, it can cause rapid fire spread in the event of fire. Probably such a short time to ignition is caused by varnishes applied to the surface of the leather.

## Figures and Tables

**Table 1:** Elementary composition of tested leathers and percentages of C, H, N, and S.

Material Name	Elementary Composition			
	%C	%H	%N	%S
Grain bovine leather	45.75	6.56	8.56	1.4
Lacquered bovine leather	44.92	6.02	13.25	1.11
Nubuck leather	46.56	6.21	8.02	1.2
Velour bovine leather	41.49	6.25	13.09	0.86

**Table 2:** TGA results of samples.

Tested Parameters	Grain Bovine Leather	Lacquered Bovine Leather	Nubuck Leather	Velour Bovine Leather
Temperature of thermal-decomposition beginning (°C)	282	275	302	281
Temperature of 50% mass loss (°C)	320	334	313	343
Final temperature of thermal decomposition (°C)	353	339	324	405
Mass residue after combustion (%)	9.1	7.3	5.5	6.5
Maximum mass-loss rate (%/min)	174	129.2	147.1	93.1
Temperature of maximum mass-loss rate (°C)	320	332	313	382

**Table 3:** Averages values of maximum specific optical density value D_s,max_, time to reach D_s,max_ [s], specific optical density value after 4 min D_s,4_, and area under the specific optical density curve during the first 4 min V_OF4_ [min].

Sample Name	D_s,max_ [-]	Time to Achieve D_s,max_ [s]	D_s,4_ [-]	V_OF4_ [min]
Combustion Without Pilot Flame (25 kW/m^2^)				
Grain bovine leather	312	300	296	694
Velour bovine leather	297	372	273	555
Nubuck leather	367	428	243	393
Lacquered bovine leather	220	442	202	512
Combustion with Pilot Flame (25 kW/m^2^)				
Grain bovine leather	38	190	32	104
Velour bovine leather	138	127	122	314
Nubuck leather	146	340	132	286
Lacquered bovine leather	68	78	58	198
Combustion Without Pilot Flame (50 kW/m^2^)				
Grain bovine leather	180	70	146	543
Velour bovine leather	166	87	143	509
Nubuck leather	256	178	243	688
Lacquered bovine leather	117	62	94	350

## Data Availability

The original contributions presented in this study are included in the article. Further inquiries can be directed to the corresponding authors.
